# Aqua­bis(5-methyl­pyrazine-2-carboxyl­ato)zinc(II) trihydrate

**DOI:** 10.1107/S1600536809031778

**Published:** 2009-08-19

**Authors:** Yong-Ming Cui, Jin Li, Xian Zhang, Qing-Fu Zeng

**Affiliations:** a Engineering Research Center for Clean Production of Textile Dyeing and Printing, Ministry of Education, Wuhan 430073, People’s Republic of China

## Abstract

In the title compound, [Zn(C_6_H_5_N_2_O_2_)_2_(H_2_O)]·3H_2_O, the Zn^II^ centre is five-coordinated by two *O*,*N*-bidentate Schiff base ligands and one O atom from a water mol­ecule in a slightly distorted square-pyramidal geometry. In the crystal, the complex and uncoordinated water mol­ecules are linked by O—H⋯O, O—H⋯N and C—H⋯O hydrogen bonds, forming a three-dimensional network.

## Related literature

For background to the mol­ecular architecture and biological activity of benzoic acid–metal complexes, see: Cheng *et al.* (2006 ▶); Yang *et al.* (2004 ▶). For reference structural data, see: Allen *et al.* (1987 ▶).
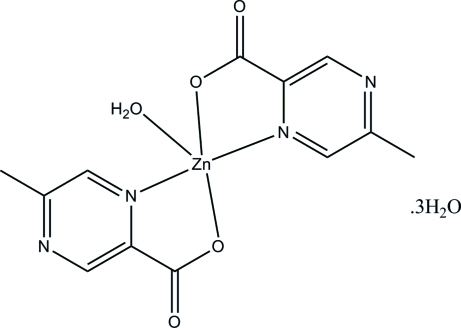


## Experimental

### 

#### Crystal data

[Zn(C_6_H_5_N_2_O_2_)_2_(H_2_O)]·3H_2_O
*M* *_r_* = 411.67
Triclinic, 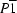

*a* = 8.134 (4) Å
*b* = 10.492 (5) Å
*c* = 10.982 (5) Å
α = 66.61 (2)°
β = 81.85 (2)°
γ = 78.33 (2)°
*V* = 840.3 (7) Å^3^
*Z* = 2
Mo *K*α radiation
μ = 1.51 mm^−1^
*T* = 296 K
0.32 × 0.28 × 0.23 mm

#### Data collection

Enraf–Nonius CAD-4 diffractometer
Absorption correction: ψ scan (North *et al.*, 1968 ▶) *T*_min_ = 0.644, *T*_max_ = 0.723
4392 measured reflections
2923 independent reflections
2539 reflections with *I* > 2σ(*I*)
*R*_int_ = 0.018
3 standard reflections every 200 reflections intensity decay: 1%

#### Refinement

*R*[*F* ^2^ > 2σ(*F* ^2^)] = 0.038
*wR*(*F* ^2^) = 0.109
*S* = 1.05
2923 reflections
260 parameters
12 restraints
H atoms treated by a mixture of independent and constrained refinement
Δρ_max_ = 0.43 e Å^−3^
Δρ_min_ = −0.68 e Å^−3^

### 

Data collection: *CAD-4 Software* (Enraf–Nonius, 1989 ▶); cell refinement: *CAD-4 Software*; data reduction: *XCAD4* (Harms & Wocadlo, 1995 ▶); program(s) used to solve structure: *SHELXS97* (Sheldrick, 2008 ▶); program(s) used to refine structure: *SHELXL97* (Sheldrick, 2008 ▶); molecular graphics: *SHELXTL* (Sheldrick, 2008 ▶); software used to prepare material for publication: *SHELXTL*.

## Supplementary Material

Crystal structure: contains datablocks global, I. DOI: 10.1107/S1600536809031778/hb5038sup1.cif

Structure factors: contains datablocks I. DOI: 10.1107/S1600536809031778/hb5038Isup2.hkl

Additional supplementary materials:  crystallographic information; 3D view; checkCIF report

## Figures and Tables

**Table 1:** Selected bond lengths (Å)

Zn1—N1	1.989 (2)
Zn1—N3	1.985 (2)
Zn1—O2	1.951 (2)
Zn1—O4	1.957 (2)
Zn1—O5	2.245 (3)

**Table 2:** Hydrogen-bond geometry (Å, °)

*D*—H⋯*A*	*D*—H	H⋯*A*	*D*⋯*A*	*D*—H⋯*A*
C9—H9⋯O1^i^	0.93	2.35	3.204 (4)	153
C3—H3⋯O3^ii^	0.93	2.36	3.255 (4)	162
O8—H8*B*⋯N4^ii^	0.832 (10)	2.30 (2)	3.044 (4)	150 (3)
O6—H6*E*⋯O7	0.841 (10)	2.092 (11)	2.932 (4)	177 (4)
O6—H6*D*⋯O8	0.839 (10)	1.924 (14)	2.755 (4)	171 (4)
O8—H8*A*⋯O1	0.835 (10)	1.953 (13)	2.781 (4)	171 (4)
O7—H7*B*⋯O3^iii^	0.836 (10)	1.996 (18)	2.797 (3)	160 (4)
O7—H7*A*⋯N2^iv^	0.839 (10)	2.185 (18)	2.977 (4)	157 (3)
O5—H5*B*⋯O6^v^	0.835 (10)	1.924 (11)	2.754 (4)	172 (3)
O5—H5*A*⋯O7^vi^	0.830 (10)	2.034 (11)	2.861 (4)	175 (4)

Symmetry codes: (i) 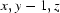; (ii) 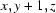; (iii) 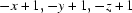; (iv) 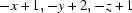; (v) 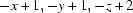; (vi) 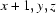.

**Fig. 1.**
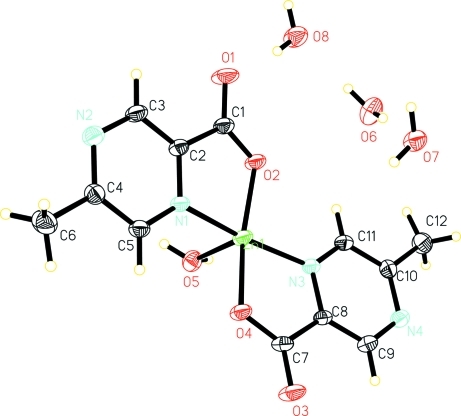
The molecular structure of (I) showing 30% probability displacement ellipsoids.

## supplementary crystallographic information

### Comment

There has been much research interest in benzoic acid metal complexes due to their molecular architectures and biological activities (Cheng *et al.*, 2006; Yang *et al.*, 2004). In this work, we report here the crystal structure of the title compound, (I). In (I), all bond lengths are within normal ranges (Allen *et al.*, 1987) (Fig. 1). The Zn^II^ atom is five-coordinated by two O and two N atoms from the two Schiff base ligands and one O from the water molecule, forming a slightly distorted square pyramid coordination (Table 1). The mononuclear complex interacts with the solvent water molecules to form a three-dimensional network (Table 2).

### Experimental

A mixture of 5-methylpyrazine-2-carboxylic acid (276 mg, 2 mmol) and Zn(NO_3_)_2_.6H_2_O (1 mmol, 271 mg) in methanol (10 ml) was stirred for 3 h. After keeping the filtrate in air for 7 d, colourless blocks of (I) were formed.

### Refinement

All H atoms were positioned geometrically (C—H = 0.93 Å for the aromatic H atoms and C—H = 0.96 Å for the aliphatic H atoms) and were refined as riding, with *U*_iso_(H) = 1.2*U*_eq_(C) and *U*_iso_(H) = 1.2*U*_eq_(N).

### Crystal data

**Table:**

[Zn(C_6_H_5_N_2_O_2_)_2_(H_2_O)]·3H_2_O	*Z* = 2
*M**_r_* = 411.67	*F*(000) = 424
Triclinic, *P*1	*D*_x_ = 1.627 Mg m^−^^3^
Hall symbol: -P 1	Mo *K*α radiation, λ = 0.71073 Å
*a* = 8.134 (4) Å	Cell parameters from 25 reflections
*b* = 10.492 (5) Å	θ = 9–12°
*c* = 10.982 (5) Å	µ = 1.51 mm^−^^1^
α = 66.61 (2)°	*T* = 296 K
β = 81.85 (2)°	Block, colourless
γ = 78.33 (2)°	0.32 × 0.28 × 0.23 mm
*V* = 840.3 (7) Å^3^	

### Data collection

**Table:**

Enraf–Nonius CAD-4 diffractometer	2539 reflections with *I* > 2σ(*I*)
Radiation source: fine-focus sealed tube	*R*_int_ = 0.018
graphite	θ_max_ = 25.0°, θ_min_ = 2.0°
ω/2θ scans	*h* = −9→9
Absorption correction: ψ scan (North *et al.*, 1968)	*k* = −12→12
*T*_min_ = 0.644, *T*_max_ = 0.723	*l* = −13→13
4392 measured reflections	3 standard reflections every 200 reflections
2923 independent reflections	intensity decay: 1%

### Refinement

**Table:**

Refinement on *F*^2^	Primary atom site location: structure-invariant direct methods
Least-squares matrix: full	Secondary atom site location: difference Fourier map
*R*[*F*^2^ > 2σ(*F*^2^)] = 0.038	Hydrogen site location: inferred from neighbouring sites
*wR*(*F*^2^) = 0.109	H atoms treated by a mixture of independent and constrained refinement
*S* = 1.05	*w* = 1/[σ^2^(*F*_o_^2^) + (0.0629*P*)^2^ + 0.6023*P*] where *P* = (*F*_o_^2^ + 2*F*_c_^2^)/3
2923 reflections	(Δ/σ)_max_ = 0.016
260 parameters	Δρ_max_ = 0.43 e Å^−^^3^
12 restraints	Δρ_min_ = −0.68 e Å^−^^3^

### Special details

**Table:**

Geometry. All e.s.d.'s (except the e.s.d. in the dihedral angle between two l.s. planes) are estimated using the full covariance matrix. The cell e.s.d.'s are taken into account individually in the estimation of e.s.d.'s in distances, angles and torsion angles; correlations between e.s.d.'s in cell parameters are only used when they are defined by crystal symmetry. An approximate (isotropic) treatment of cell e.s.d.'s is used for estimating e.s.d.'s involving l.s. planes.
Refinement. Refinement of *F*^2^ against ALL reflections. The weighted *R*-factor *wR* and goodness of fit *S* are based on *F*^2^, conventional *R*-factors *R* are based on *F*, with *F* set to zero for negative *F*^2^. The threshold expression of *F*^2^ > σ(*F*^2^) is used only for calculating *R*-factors(gt) *etc*. and is not relevant to the choice of reflections for refinement. *R*-factors based on *F*^2^ are statistically about twice as large as those based on *F*, and *R*- factors based on ALL data will be even larger.

### Fractional atomic coordinates and isotropic or equivalent isotropic displacement parameters (Å^2^)

**Table:**

	*x*	*y*	*z*	*U*_iso_*/*U*_eq_	
C1	0.6126 (4)	0.8388 (3)	0.6598 (3)	0.0398 (8)	
C2	0.7345 (4)	0.8625 (3)	0.5379 (3)	0.0328 (7)	
C3	0.7920 (4)	0.9875 (3)	0.4653 (3)	0.0402 (8)	
H3	0.7551	1.0635	0.4912	0.048*	
C4	0.9502 (4)	0.8932 (3)	0.3229 (3)	0.0371 (7)	
C5	0.8939 (4)	0.7652 (3)	0.3960 (3)	0.0358 (7)	
H5	0.9310	0.6891	0.3703	0.043*	
C6	1.0683 (5)	0.9107 (4)	0.2018 (4)	0.0555 (10)	
H6A	1.0163	0.9832	0.1257	0.083*	
H6B	1.0947	0.8237	0.1881	0.083*	
H6C	1.1699	0.9363	0.2139	0.083*	
C7	0.6723 (4)	0.3717 (3)	0.5483 (3)	0.0350 (7)	
C8	0.5573 (4)	0.3445 (3)	0.6745 (3)	0.0315 (7)	
C9	0.4702 (4)	0.2334 (3)	0.7297 (3)	0.0387 (7)	
H9	0.4846	0.1673	0.6908	0.046*	
C10	0.3477 (4)	0.3130 (4)	0.8926 (3)	0.0381 (7)	
C11	0.4392 (4)	0.4242 (3)	0.8389 (3)	0.0362 (7)	
H11	0.4281	0.4886	0.8793	0.043*	
C12	0.2293 (5)	0.2978 (4)	1.0126 (4)	0.0533 (10)	
H12A	0.1158	0.3146	0.9883	0.080*	
H12B	0.2421	0.3647	1.0487	0.080*	
H12C	0.2539	0.2042	1.0781	0.080*	
H5A	0.964 (4)	0.540 (3)	0.750 (3)	0.040 (10)*	
H7A	0.070 (5)	0.7757 (14)	0.720 (4)	0.056 (12)*	
H8A	0.406 (6)	0.908 (4)	0.855 (3)	0.090 (18)*	
H5B	0.875 (4)	0.438 (3)	0.8366 (14)	0.048 (11)*	
H7B	0.175 (4)	0.675 (4)	0.683 (3)	0.076 (15)*	
H8B	0.329 (5)	0.985 (2)	0.929 (3)	0.049 (12)*	
H6D	0.259 (6)	0.734 (3)	0.977 (4)	0.088 (18)*	
H6E	0.183 (5)	0.674 (4)	0.915 (2)	0.065 (14)*	
N1	0.7865 (3)	0.7518 (3)	0.5033 (2)	0.0317 (6)	
N2	0.9005 (4)	1.0029 (3)	0.3579 (3)	0.0417 (7)	
N3	0.5426 (3)	0.4391 (3)	0.7298 (2)	0.0318 (6)	
N4	0.3644 (4)	0.2175 (3)	0.8390 (3)	0.0405 (7)	
O1	0.5584 (4)	0.9371 (3)	0.6954 (3)	0.0624 (8)	
O2	0.5775 (3)	0.7154 (2)	0.7156 (2)	0.0423 (6)	
O3	0.6850 (4)	0.2940 (2)	0.4880 (2)	0.0514 (7)	
O4	0.7438 (3)	0.4786 (2)	0.5142 (2)	0.0394 (5)	
O5	0.9020 (3)	0.4833 (3)	0.7568 (2)	0.0449 (6)	
O6	0.2178 (4)	0.6625 (3)	0.9876 (3)	0.0635 (8)	
O7	0.0982 (4)	0.6891 (3)	0.7377 (3)	0.0494 (6)	
O8	0.3459 (4)	0.9068 (3)	0.9238 (3)	0.0654 (8)	
Zn1	0.68363 (5)	0.58672 (4)	0.62838 (4)	0.03747 (16)	

### Atomic displacement parameters (Å^2^)

**Table:**

	*U*^11^	*U*^22^	*U*^33^	*U*^12^	*U*^13^	*U*^23^
C1	0.0472 (19)	0.0338 (17)	0.0442 (18)	−0.0088 (14)	0.0072 (15)	−0.0233 (15)
C2	0.0402 (17)	0.0294 (15)	0.0330 (15)	−0.0068 (13)	−0.0022 (13)	−0.0157 (13)
C3	0.051 (2)	0.0308 (16)	0.0444 (18)	−0.0087 (14)	0.0016 (15)	−0.0211 (15)
C4	0.0361 (17)	0.0369 (17)	0.0363 (16)	−0.0046 (13)	−0.0006 (13)	−0.0131 (14)
C5	0.0402 (18)	0.0325 (16)	0.0380 (17)	−0.0037 (13)	−0.0005 (14)	−0.0187 (14)
C6	0.059 (2)	0.048 (2)	0.050 (2)	−0.0061 (18)	0.0150 (18)	−0.0161 (18)
C7	0.0468 (18)	0.0249 (15)	0.0339 (16)	−0.0017 (13)	−0.0005 (14)	−0.0146 (13)
C8	0.0359 (16)	0.0247 (14)	0.0368 (16)	−0.0031 (12)	−0.0015 (13)	−0.0158 (13)
C9	0.0465 (19)	0.0318 (16)	0.0431 (18)	−0.0077 (14)	−0.0030 (15)	−0.0190 (15)
C10	0.0327 (17)	0.0417 (18)	0.0410 (17)	−0.0068 (13)	0.0010 (13)	−0.0178 (15)
C11	0.0392 (17)	0.0352 (16)	0.0406 (17)	−0.0054 (13)	0.0015 (14)	−0.0226 (14)
C12	0.053 (2)	0.062 (2)	0.050 (2)	−0.0182 (18)	0.0151 (17)	−0.0278 (19)
N1	0.0369 (14)	0.0270 (12)	0.0339 (13)	−0.0041 (10)	−0.0003 (11)	−0.0158 (11)
N2	0.0494 (17)	0.0327 (14)	0.0427 (15)	−0.0095 (12)	0.0032 (13)	−0.0147 (12)
N3	0.0352 (14)	0.0287 (13)	0.0349 (13)	−0.0047 (10)	0.0007 (11)	−0.0169 (11)
N4	0.0444 (16)	0.0356 (14)	0.0450 (16)	−0.0121 (12)	0.0025 (13)	−0.0181 (13)
O1	0.092 (2)	0.0378 (13)	0.0634 (17)	−0.0178 (13)	0.0312 (15)	−0.0344 (13)
O2	0.0563 (14)	0.0331 (12)	0.0444 (13)	−0.0164 (10)	0.0166 (11)	−0.0243 (10)
O3	0.0831 (19)	0.0376 (13)	0.0436 (13)	−0.0201 (12)	0.0148 (13)	−0.0273 (11)
O4	0.0550 (14)	0.0322 (12)	0.0372 (12)	−0.0144 (10)	0.0103 (10)	−0.0205 (10)
O5	0.0494 (15)	0.0442 (14)	0.0403 (14)	−0.0148 (11)	0.0000 (11)	−0.0127 (12)
O6	0.079 (2)	0.071 (2)	0.0412 (15)	−0.0360 (17)	0.0014 (14)	−0.0121 (14)
O7	0.0609 (17)	0.0442 (15)	0.0444 (14)	−0.0189 (12)	0.0128 (12)	−0.0185 (12)
O8	0.080 (2)	0.0525 (18)	0.0684 (19)	−0.0268 (15)	0.0282 (16)	−0.0311 (15)
Zn1	0.0488 (3)	0.0304 (2)	0.0392 (2)	−0.01200 (16)	0.00646 (16)	−0.01988 (17)

### Geometric parameters (Å, °)

**Table:**

C1—O1	1.224 (4)	C10—N4	1.327 (4)
C1—O2	1.266 (4)	C10—C11	1.394 (5)
C1—C2	1.516 (4)	C10—C12	1.492 (5)
C2—N1	1.334 (4)	C11—N3	1.336 (4)
C2—C3	1.373 (4)	C11—H11	0.9300
C3—N2	1.345 (4)	C12—H12A	0.9600
C3—H3	0.9300	C12—H12B	0.9600
C4—N2	1.325 (4)	C12—H12C	0.9600
C4—C5	1.395 (5)	Zn1—N1	1.989 (2)
C4—C6	1.497 (5)	Zn1—N3	1.985 (2)
C5—N1	1.341 (4)	Zn1—O2	1.951 (2)
C5—H5	0.9300	Zn1—O4	1.957 (2)
C6—H6A	0.9600	Zn1—O5	2.245 (3)
C6—H6B	0.9600	O5—H5A	0.830 (10)
C6—H6C	0.9600	O5—H5B	0.835 (10)
C7—O3	1.221 (4)	O6—H6D	0.839 (10)
C7—O4	1.267 (4)	O6—H6E	0.841 (10)
C7—C8	1.518 (4)	O7—H7A	0.839 (10)
C8—N3	1.334 (4)	O7—H7B	0.836 (10)
C8—C9	1.370 (4)	O8—H8A	0.835 (10)
C9—N4	1.347 (4)	O8—H8B	0.832 (10)
C9—H9	0.9300		
			
O1—C1—O2	126.4 (3)	N3—C11—H11	119.7
O1—C1—C2	118.7 (3)	C10—C11—H11	119.7
O2—C1—C2	115.0 (3)	C10—C12—H12A	109.5
N1—C2—C3	120.3 (3)	C10—C12—H12B	109.5
N1—C2—C1	115.6 (3)	H12A—C12—H12B	109.5
C3—C2—C1	124.1 (3)	C10—C12—H12C	109.5
N2—C3—C2	121.7 (3)	H12A—C12—H12C	109.5
N2—C3—H3	119.2	H12B—C12—H12C	109.5
C2—C3—H3	119.2	C2—N1—C5	118.9 (3)
N2—C4—C5	121.2 (3)	C2—N1—Zn1	110.7 (2)
N2—C4—C6	118.0 (3)	C5—N1—Zn1	130.3 (2)
C5—C4—C6	120.8 (3)	C4—N2—C3	117.8 (3)
N1—C5—C4	120.0 (3)	C8—N3—C11	118.4 (3)
N1—C5—H5	120.0	C8—N3—Zn1	111.5 (2)
C4—C5—H5	120.0	C11—N3—Zn1	130.1 (2)
C4—C6—H6A	109.5	C10—N4—C9	117.6 (3)
C4—C6—H6B	109.5	C1—O2—Zn1	115.0 (2)
H6A—C6—H6B	109.5	C7—O4—Zn1	115.3 (2)
C4—C6—H6C	109.5	Zn1—O5—H5A	112 (2)
H6A—C6—H6C	109.5	Zn1—O5—H5B	114 (2)
H6B—C6—H6C	109.5	H5A—O5—H5B	110.6 (17)
O3—C7—O4	126.2 (3)	H6D—O6—H6E	108.5 (17)
O3—C7—C8	119.0 (3)	H7A—O7—H7B	109.8 (17)
O4—C7—C8	114.8 (3)	H8A—O8—H8B	110.3 (18)
N3—C8—C9	120.6 (3)	O2—Zn1—O4	166.16 (10)
N3—C8—C7	115.2 (3)	O2—Zn1—N3	95.36 (10)
C9—C8—C7	124.2 (3)	O4—Zn1—N3	83.22 (9)
N4—C9—C8	121.7 (3)	O2—Zn1—N1	83.64 (9)
N4—C9—H9	119.2	O4—Zn1—N1	95.00 (10)
C8—C9—H9	119.2	N3—Zn1—N1	168.45 (10)
N4—C10—C11	121.0 (3)	O2—Zn1—O5	97.35 (10)
N4—C10—C12	118.2 (3)	O4—Zn1—O5	96.49 (10)
C11—C10—C12	120.8 (3)	N3—Zn1—O5	94.98 (10)
N3—C11—C10	120.6 (3)	N1—Zn1—O5	96.55 (10)

### Hydrogen-bond geometry (Å, °)

**Table:**

*D*—H···*A*	*D*—H	H···*A*	*D*···*A*	*D*—H···*A*
C9—H9···O1^i^	0.93	2.35	3.204 (4)	153
C3—H3···O3^ii^	0.93	2.36	3.255 (4)	162
O8—H8B···N4^ii^	0.83 (1)	2.30 (2)	3.044 (4)	150 (3)
O6—H6E···O7	0.84 (1)	2.09 (1)	2.932 (4)	177 (4)
O6—H6D···O8	0.84 (1)	1.92 (1)	2.755 (4)	171 (4)
O8—H8A···O1	0.84 (1)	1.95 (1)	2.781 (4)	171 (4)
O7—H7B···O3^iii^	0.84 (1)	2.00 (2)	2.797 (3)	160 (4)
O7—H7A···N2^iv^	0.84 (1)	2.19 (2)	2.977 (4)	157 (3)
O5—H5B···O6^v^	0.84 (1)	1.92 (1)	2.754 (4)	172 (3)
O5—H5A···O7^vi^	0.83 (1)	2.03 (1)	2.861 (4)	175 (4)

Symmetry codes: (i) *x*, *y*−1, *z*; (ii) *x*, *y*+1, *z*; (iii) −*x*+1, −*y*+1, −*z*+1; (iv) −*x*+1, −*y*+2, −*z*+1; (v) −*x*+1, −*y*+1, −*z*+2; (vi) *x*+1, *y*, *z*.
